# Evaluation of biochemical and reproductive biomarkers on gestational period in immunosuppressed Wistar rats with Cyclosporin

**DOI:** 10.1186/1758-5996-7-S1-A68

**Published:** 2015-11-11

**Authors:** Aline Zanatta Schavinski, Nagilla Orleanne Lima do Carmo, Elisangela Miranda de Jesus Lisboa, Gilsielle Benicio Jaco, Betina Beatriz Mielke, Madileine Francely Américo, Luciana Aparecida Cora, Maria do Carmo Borges Teixeira, Kleber Eduardo de Campos

**Affiliations:** 1 Universidade Federal de Mato Grosso, Barra do Garças, Brazil

## Background

The pregnancy period involves a lot of biological changes, and the use of medication must be taken with extremely caution, especially because of its implications for maternal and fetal health, involving biochemical and reproductive functions in maternal organism. Also, the Cyclosporin A (CLP) is used to avoid possible rejection of transplanted tissues, being one of most immunosuppressant used.

## Objective

The objective of this study was to evaluate the effects of immunosuppressive therapy with Cyclosporine A in reproductive and biochemical profile in pregnant Wistar rats.

## Materials and methods

The rats were randomized into three groups CONT (treated with tap water), CLP1 (treated with Cyclosporin A 15 mg/kg before and during pregnancy) and CLP2 (treated with Cyclosporin A 15 mg/kg before pregnancy), n=8 per group. The body weight, food and water intake, and glucose were measured weekly. On the 17th day of pregnancy it was evaluated the oral glucose tolerance test (OGTT). The reproductive parameters were determined by fertility indices, pregnancy and childbirth. On the last day of pregnancy (day 21), serum biochemical analysis were performed (total protein, cholesterol, triglycerides from high density lipoprotein (HDL) cholesterol and very low density lipoprotein (VLDL). The statistical significance was considered p<0.05.

## Results

It was observed a progressive increase in body weight during the pregnancy in all groups compared to the first day. In addition, the continued use of this drug decreased food intake and slightly blood glucose. The reproductive parameters have not changed. The glycemic curve generated by OGTT show the timepoint of 120 min of CLP1 group was increased related to other groups. In biochemical evaluation, serum cholesterol and HDL were unchanged, but the treatment with the drug led increase in protein concentration (CLP2), triglyceride and VLDL (CLP1) and also an increase in ALT activity, AST and reduced (in both groups).

## Conclusion

The use of Cyclosporine A during pregnancy has some security for weight gain, lipid metabolism and also maternal insulin action in maternal organism.

